# Abstinence beliefs in early adolescence and sexual risk behavior two years later

**DOI:** 10.1002/jad.12365

**Published:** 2024-06-22

**Authors:** Shristi Bhochhibhoya, Briana Edison, Elizabeth R. Baumler, Christine M. Markham, Susan T. Emery, Melissa F. Peskin, Ross Shegog, Robert C. Addy, Jeff R. Temple, Dennis E. Reidy

**Affiliations:** 1 Department of Health, Human Performance & Recreation, University of Arkansas, Fayetteville, North Carolina, USA; 2 School of Public Health & Center for Research on Interpersonal Violence Georgia State University, Atlanta, Georgia, USA; 3 School of Behavioral Health Sciences, University of Texas Health Science Center at Houston, Houston, Texas, USA; 4 School of Public Health, University of Texas Health Science Center at Houston, Houston, Texas, USA

**Keywords:** abstinence beliefs, beliefs to delay sex, sexual risk behaviors

## Abstract

**Introduction:**

The United States has the highest teen pregnancy rate and sexually transmitted infection rates among developed countries. One common approach that has been implemented to reduce these rates is abstinence-only-until-marriage programs that advocate for delaying sexual intercourse until marriage. These programs focus on changing adolescents’ beliefs toward abstinence until marriage; however, it is unclear whether adolescents’ beliefs about abstinence predict their sexual behavior, including sexual risk behavior (SRB). An alternative approach may be encouraging youth to delay their sexual debut until they reach the age of maturity, but not necessarily until marriage.

**Methods:**

To address this question, we compare the longitudinal association between abstinence beliefs (i.e., abstaining completely until marriage) and beliefs about delayed sexual debut with subsequent SRB 24 months later. The harmonized data set included 4620 (58.2% female, *M*_age_ = 13.0, SD_age_ = 0.93) participants from three randomized controlled trials attending 44 schools in the southern United States. Negative binomial regressions were employed to examine the association of abstinence until marriage beliefs and beliefs regarding delaying sex with SRB.

**Results:**

We identified that beliefs supporting delaying sex until an age of maturity were associated with lower odds of engaging in SRB, such as having multiple sex partners and frequency of condomless sex, for both sexes. However, stronger abstinence beliefs had no significant associations with all SRB outcomes.

**Conclusions:**

Findings suggest prevention programming that focuses on encouraging youth to delay sex until an appropriate age of maturity may be more effective at preventing SRB and consequent negative sexual health outcomes.

## 1 | INTRODUCTION

Abstinence-only-until-marriage (AOUM) education is defined as any educational or motivational program that (1) teaches that social, psychological, and health gains are realized by abstaining from sexual activity; (2) teaches that abstinence is the only way to avoid unwanted pregnancy and STIs; (3) teaches that sexual activity outside of marriage has harmful consequences; and (4) teaches that having children out-of-wedlock is harmful to children and parents (Santelli et al., 2006; Young & Penhollow, 2006). The US federal government started AOUM programs in 1981 as a means of combatting the highest rates of teen pregnancy and sexually transmitted infections (STIs) among developed nations (Aral et al., 2007; Sedgh et al., 2015). In 1996, a provision was added to the Welfare Reform Act to fund and promote AOUM education programs that emphasize refraining from any nonmarital sexual activity (Advocates for Youth, 2007; Santelli et al., 2017). To further expand AOUM programs, the US Congress added a new funding mechanism, the “Sexual Risk Avoidance Education” (SRAE) program, in fiscal year 2016. Since the program’s inception, the government has spent $2 billion on AOUM programs in the United States. Funding in excess of $75 million annually has been invested by the Title V grant program to implement AOUM programs focused primarily on refraining from all sex until marriage while restricting access to information about contraception and safe sexual practices (Guttmacher Institute, 2021).

Despite public and fiscal support, empirical support for AOUM programs is mixed. Several evaluations have found that these programs are not evidence-based, may interfere with complete and accurate health information, and have little to no impact on the sexual behavior of youth (Denny & Young, 2006; Santelli et al., 2017). For example, an evaluation of AOUM programs conducted in 10 states confirmed no significant reduction in teens’ sexual behavior at follow-up and 3–17 months after the completion of the program (Hauser, 2004). Similarly, no significant impacts were observed in teen sexual activity and rates of unprotected sex among participants receiving AOUM education when compared to the control group (Trenholm et al., 2007). Conversely, a recent meta-analysis (Jeynes, 2020) reported that AOUM programs did have a significant but small effect on sexual behavior (*d* = .22–.38). It is important to acknowledge that this analysis appears to include studies without control groups as well as cross-sectional, correlational, and retrospective designs. The studies included in the meta-analysis are not provided, making it difficult to judge the appropriateness of their inclusion in the analysis or interpret the results as a whole. Perhaps more importantly, this study (like most others) does not address the mechanism by which AOUM programs do or do not affect sexual behavior.

An emerging argument against programs that only teach abstinence until marriage is the steady rise in the age of first marriages (Santelli et al., 2017; Wellings et al., 2006). Asking youth and young adults to abstain from sexual intercourse until marriage may be increasingly impractical and potentially ineffective and counterproductive. Currently, the gap between the age of first intercourse and first marriage is 11.7 years for men and 8.7 years for women (Finer & Philbin, 2014). Thus, putting an exclusive focus on marriage to initiate sexual activity in sex education during middle and high school could negatively impact adolescent’s willingness to participate in safe sex behaviors. Alternatively, borrowing from harm-reduction strategies, we can educate adolescents to postpone sexual activity until they are mature, when they can better decide about relationships and have accurate information about contraception. To better equip adolescents with the necessary knowledge, health professionals have overwhelmingly supported comprehensive sex education, which includes abstinence, delayed sexual debut, education about contraception, and access to sexual and reproductive health information (Santelli et al., 2017).

This knowledge of sexual health is particularly important for adolescents as they are susceptible to engaging in sexual risk behavior (SRB), such as infrequent use of condoms or having multiple partners (O’Donnell et al., 2001; Scott et al., 2011). These behavioral patterns during adolescence may continue throughout adulthood and exacerbate the risk of adverse health outcomes such as sexually transmitted infections and unwanted pregnancy (Centers for Disease Control and Prevention [CDC], n.d.). Given the profound physical, social, and financial impacts on the lives of adolescents, SRB among adolescents is a critical public health concern.

Beliefs about when it is appropriate for initiation of sexual activity, whether that is based on marriage or maturity, may influence the onset of sexual behavior (Buhi et al., 2011; Gray et al., 2008). However, there are no clear data suggesting beliefs about abstinence until marriage, which are the focus of federally funded AOUM programs, influence adolescents’ initiation of sexual behavior. One study (Akers et al., 2011) compared the predictive utility of adolescents’ personal beliefs about abstinence and compared them to their perceptions of peers’ and families’ beliefs about sex. They found that the personal beliefs about abstinence were the strongest predictor of oral and vaginal sex. However, their measure of abstinence conflates “waiting to have sex until one is older, in love, or married.” Thus, it is unclear which beliefs drove this association. Differentiating beliefs regarding “abstinence until marriage” from beliefs related to “delaying sex until the age of maturity” is important as these concepts have different connotations for adolescents who are developing physically and emotionally and their decisions about sex are evolving.

To fill this gap in the literature, we explored the individual influence of adolescents’ beliefs regarding abstinence until marriage and beliefs regarding delaying sex until maturity on engagement in SRB during adolescence. The goal of this study was to explore the longitudinal association of adolescents’ beliefs about abstinence until marriage and beliefs about delayed sexual debut on sexual outcomes. Specifically, we assessed whether abstinence beliefs and beliefs about delayed sex among students in middle school (7th and 8th graders) were related to a series of SRBs 2 years later when they were in high school (9th and 10th graders).

## 2 | METHODS

### 2.1 | Participants and procedures

We used an integrated data analysis (IDA) framework to pool student-level data across three large randomized clinical trials of adolescents (Markham et al., 2012; Peskin et al., 2015; Tortolero et al., 2010). Data were collected between 2004 and 2012 using an audio computer-assisted self-interview on laptop computers in each randomized clinical trial. All RCTs were evaluations of distinct programs intended to prevent SRB and subsequent pregnancy and sexually transmitted infections among adolescents. For more details regarding the sample, refer to the original publications (Markham et al., 2012; Peskin et al., 2015; Tortolero et al., 2010). The IDA framework provides the ability to create larger samples, allowing assessment of program effects on low-frequency outcomes and within subpopulations of the sample where statistical power is limited within the context of any single study. Statistically, base rates for SRB are relatively low in the population of young adolescents (Underwood et al., 2020). Thus, IDA allows us to increase sample size and statistical power for analysis of the specific outcomes presented herein. A set of common quantitative measures across the study datasets was identified. For any given measure, the optimal situation was when all studies used identical items regarding wording and response options. All variations in item wording and response options were resolved before the datasets were combined.

Participants were 4620 students (58.27% female participants, *n* = 2691) from 44 schools in the southwest United States. As shown in Table 1, youth were primarily racial and ethnic minorities, with a mean age of 13 at baseline (58.2% female participants). All study procedures received approval from the University of Texas Health Science Center Houston’s IRB.

### 2.2 | Measures

#### Demographics:

Youth reported their biological sex, age, race, ethnicity, and mother and father’s highest level of education.

#### Abstinence beliefs:

Three items (α = .71) assessed youths’ beliefs about abstaining from sex until marriage (i.e., “I believe having sex before marriage is wrong,” “The best way for young people to avoid an unwanted pregnancy or STD is to wait until they are married to have sex,” and “It is important to me to get married before having sex”). Using a 4-point scale, response options ranged from strongly disagree (1) to strongly agree (4).

#### Beliefs regarding delayed sexual debut:

Four items (α = .75) assessed beliefs about waiting to have sex until an older age (i.e., “I believe people my age should wait until they are older to have sex,” “I believe it is ok for people my age to have sex with a steady boyfriend or girlfriend,” “I believe it is ok for people my age to have sex as long as they use a condom,” and “I believe it is ok for people my age to have sex with a friend/casual friend”). Three of the items were recoded such that a greater score meant stronger beliefs regarding delayed sex. Using a 4-point scale, response options ranged from strongly disagree (1) to strongly agree (4).

#### SRB:

Participants reported a series of vaginal SRB at baseline and the 24-month follow-up. SRB included the number of vaginal sex partners, frequency of vaginal sex in the past 3 months, frequency of using drugs before sexual vaginal intercourse in the past 3 months, frequency of vaginal sex without a condom, and number of vaginal partners without a condom in the preceding 3 months. A 3-month reporting period was used to minimize recall bias.

## 3 | ANALYSIS

We conducted analyses in Mplus V.8.8 with robust standard errors (i.e., Huber-White sandwich estimator) to account for the clustering of youth within schools. We handled missing data via full information maximum likelihood (FIML) estimation and used negative binomial regression to account for over-dispersion in the outcome variables. Models were computed separately for male and female participants and controlled for age, race, ethnicity, intervention status (i.e., whether a student was in a treatment or control condition), and parental education. We created a latent factor for parental education with two ordinal indicators for mothers’ and fathers’ education. We used latent factor scores of the two abstinence beliefs scores in the final model. This method is superior to summing or averaging individual items because it accounts for measurement errors (McNeish & Wolf, 2020; Skrondal & Laake, 2001). Additionally, we controlled for exposure to teen pregnancy prevention interventions (Markham et al., 2012; Peskin et al., 2015; Tortolero et al., 2010) and following a fixed-effects IDA framework, we controlled study membership using effect-coded dummy variables. Finally, sexual behavior at baseline was included in all models as a covariate. Participants who were not sexually active at baseline scored zero for all SRB. Thus, all parameter estimates reflect the effects of abstinence until marriage and delayed sex beliefs for youth who had not sexually debuted at baseline.

## 4 | RESULTS

As shown in Table 2, beliefs about abstinence until marriage at baseline were not significantly associated with any subsequent SRB for both sexes. This means that an increase in the score related to abstinence beliefs is not associated with any SRB measured for both male and female participants.

Among female participants, beliefs about delaying sex at baseline was a protective factor against all measured SRB. That is, the higher the score female participants had for beliefs about delaying their sexual debut, the less likely they were to engage in all SRB measured in this study. Female participants with stronger beliefs regarding delayed sexual debut were more likely to have fewer lifetime sexual partners (IRR = 0.34), decreased frequency of sex (IRR = 0.24), decreased frequency of use of drugs/alcohol before sex (IRR = 0.08), fewer unprotected vaginal sex (IRR = 0.14), fewer number of partners (IRR = 0.30) and fewer partners with unprotected sex (IRR = 0.19).

Among male participants, except for the frequency of condomless sex and the number of partners with condomless sex, beliefs regarding delayed sexual debut were also protective against subsequent SRB. Table 2 shows that the adolescent boys with stronger beliefs regarding delayed sexual debut were more likely to have fewer lifetime sexual partners (IRR = 0.54), decreased frequency of sex (IRR = 0.58), decreased frequency of use of drugs/alcohol before sex (IRR = 0.03), and fewer number of partners (IRR = 0.36).

## 5 | DISCUSSION

Using a large ethnically diverse sample of young adolescents, we found that beliefs about abstinence until marriage were not significantly associated with subsequent SRB, including the number of sexual partners and the frequency of vaginal sex. Conversely, beliefs about delaying sex until an age of maturity were predictive of engaging in less SRB, which are themselves linked to unintended teen pregnancies and STIs. These findings remained even after controlling for baseline behavior and several demographic factors. With minor exceptions, the pattern of results was similar for male and female participants.

Our findings suggest AOUM programs may not be an effective primary prevention strategy. Indeed, in this first study to prospectively compare the effect of beliefs about abstinence until marriage versus delaying sexual debut on subsequent SRB, we found that the latter approach may be more pertinent to reducing SRB among adolescents. It is likely that the more stringent messaging of AOUM programs is less well-received by adolescent populations who are in a phase of sexual and physical exploration. Qualitative studies indicate that adolescents perceive abstinence-only until-marriage educational content as too restrictive, biased, and irrelevant, causing them to seek outside sources of sexual health information (Allen, 2008; Gardner, 2015; Kimmel et al., 2013). Our findings, coupled with the extant literature, suggest that a more effective strategy in preventing SRB and associated outcomes may be sex education interventions that take a harm reduction approach and include messaging to delay sexual debut until a reasonable age of maturity rather than asking adolescents to wait until marriage (Kirby, 2008; Stanger-Hall & Hall, 2011).

In most evaluations of AOUM programs, beliefs related to delayed sex or abstinence until marriage are often assessed alongside behavioral outcomes, such as participants’ history of sexual intercourse (Borawski et al., 2005; Markham et al., 2012). Often, evaluators assess changes in these beliefs utilizing pre- and postevaluations (Borawski et al., 2005; Markham et al., 2012) but have failed to assess beliefs as potential mediators for future engagement in SRB. We found that beliefs regarding abstinence until marriage are not indicative of future SRB and, thus, may not be indicative of the effectiveness of abstinence-based pregnancy and STI prevention programs. It is important to note that the AOUM message may not be effective at delaying sexual activity or increasing protective sexual behaviors. Instead, beliefs about delaying the initiation of sex until an age of maturity may serve as a better proxy for measuring the effectiveness of pregnancy and STI prevention programs. This latter notion may be especially true for prevention programs implemented in early adolescence when base rates of SRB, pregnancy, and STIs are low and constraints on time and financial resources preclude the collection of large longitudinal samples spanning multiple years of adolescence.

This study is not devoid of limitations. The accuracy of self-reported data is based on young participants’ ability to comprehend the questions, recall behaviors, and their motivation to provide accurate information (Glassman et al., 2022). Future research may seek other strategies to corroborate self-reported outcomes. Nevertheless, self-reports of adolescents’ health risk behavior are essential to public health practice (Glassman et al., 2022; Underwood et al., 2020). Additionally, while 2 years reflect a significant span of change during adolescence, our sample was only followed through 9th/10th grade. Sexual debut continues to rise over the next few years (CDC, n.d.) and the relationships between beliefs and behavior may change as youth enter the later stages of adolescence. Of course, only 3% of youth have sexual intercourse before high school, while the rate increases to 19% and 34% by 9th and 10th grade, respectively (CDC, n.d.). This would suggest these first years of high school are a critical stage for the onset of risk. Finally, although this study considered a series of SRB involving vaginal sexual intercourse, other sexual behaviors, such as oral and anal sex behaviors, were not included. Future studies may explore the association of abstinence beliefs with engagement in diverse SRB beyond vaginal sex.

## 6 | CONCLUSIONS

Our findings indicate that beliefs about delaying sexual debut until an age of maturity may be a better indicator of future sexual risk behaviors than beliefs about abstinence until marriage. Specifically, for both middle school-aged boys and girls, the beliefs regarding delayed sex were inversely linked to SRB, while the beliefs regarding abstinence until marriage had no relation. These findings suggest that (1) attitudes about delaying sexual debut may be a better proxy for examining the efficacy of sexual risk prevention programs, and (2) interventions focusing on delaying sexual debut versus abstaining from sex until marriage may be more effective at preventing unintended pregnancy and transmissions of sexually transmitted diseases.

## Figures and Tables

**TABLE 1:** Descriptive statistics of sample.

	Males (*N* = 1927)		Females (*N* = 291)	
Variables	*N* (Mean)	% (SD)	*N* (Mean)	% (SD)
Age	(13.1)	(0.95)	(12.9)	(0.9)
Race				
White	128	6.6	183	6.8
Black	709	37.0	1073	39.9
Hispanic	1,005	52.4	1366	50.8
Other	199	7.4	310	11.5
Mother’s education				
Did not complete high school	605	37.5	1004	42.3
High school or GED	420	26.1	584	24.6
Did some college or training after high school	248	15.4	356	15.0
Completed college	339	21.0	429	18.1
Father’s education				
Did not complete high school	389	42.7	584	46.8
High school or GED	232	25.5	325	26.0
Did some college or training after high school	123	13.5	147	11.8
Completed college	166	18.2	193	15.5
Intervention				
Control	1102	57.2	1567	58.2
Treatment	825	42.8	1124	41.8
Abstinence Beliefs				
Beliefs about waiting to have sex	(10.89)	(2.67)	(12.31)	(2.51)
Beliefs about abstinence until marriage	(7.67)	(2.10)	(8.46)	(2.11)

*Note*: Participants were asked to “select all that apply” for race. “Other” includes participants who identified as Asian, American Indian, and others. GED General Education Development.

**TABLE 2:** Association between abstinence until marriage beliefs and delayed sexual debut beliefs with subsequent vaginal sex behaviors using negative binomial regression.

		Males (*N* = 1927)		Females (*N* = 2691)	
Outcome variables		IRR	*p*-Value	IRR	*p*-Value
Lifetime number of partners	B_delay_	**0.54**	<**.001**	**0.34**	<**.001**
	B_abstinence_	0.77	.09	1.12	.49
Frequency of sex	B_delay_	**0.58**	**.02**	**0.24**	<**.001**
	B_abstinence_	0.62	.13	0.87	.68
Frequency of drug/alcohol use before sex	B_delay_	**0.03**	<**.001**	**0.08**	<**.001**
	B_abstinence_	2.47	.29	1.56	.41
Frequency of condomless sex	B_delay_	0.40	.08	**0.14**	<**.001**
	B_abstinence_	0.77	.64	1.01	.98
Number of partners	B_delay_	**0.36**	**.01**	**0.30**	<**.001**
	B_abstinence_	0.80	.63	0.96	.84
Number of partners without condom	B_delay_	0.59	.40	**0.19**	<**.001**
	B_abstinence_	0.27	.07	1.46	.22

*Note*: B_delay_ = Belief about waiting to have sex/delayed sexual debut; B_abstinence_ = Belief about abstinence until marriage. Separate models were run for each dependent variables (vaginal sex behaviors) controlling for age, race, parental education, intervention effects, and corresponding outcome measured at baseline. IRR, Incident Rate Ratio is used to compare the rate of increase in sexual risk behavior measured. Bold text represent significant results.

## Data Availability

The data that support the findings of this study are available from the corresponding author upon reasonable request.
